# Immune cell dynamics and cytokine regulation in cornea and transplantation

**DOI:** 10.3389/fimmu.2026.1847174

**Published:** 2026-08-04

**Authors:** Ankur Sharma, Ahmad AI Aiyan, Uday Kishore

**Affiliations:** 1 Department of Veterinary Medicine, United Arab Emirates University, Al Ain, United Arab Emirates; 2 Zayed Centre for health sciences, United Arab Emirates university, Al Ain, United Arab Emirates

**Keywords:** cornea, cytokines, complement, immune receptors, ocular immunity, transplantation

## Abstract

Immune privilege is essential for clear vision in the cornea. It assists in maintaining a strong defense against pathogens while limiting inflammation. This advantage results from a combination of physical barriers, such as the blood-eye barrier, local anti-inflammatory molecules, T cell apoptosis, resident and infiltrating immune cells, cytokine network, and humoral factors. These dynamic processes collaborate to protect the cornea and prevent tissue damage. While the development of ocular T regulatory cells is well-known, the specific mechanisms through which they suppress immune-mediated inflammation are not fully characterized. Recent advances in imaging and molecular biology have revealed the dynamic behavior of corneal immune cells and their regulatory roles in the production of cytokines and other humoral mediators. This review summarizes existing knowledge on corneal immune cell dynamics, cytokine regulation, and the mechanisms that support immune privilege and its rejection in corneal transplantation. We highlight the continuing discussions, identifying research shortcomings, and innovative therapeutic approaches targeting these pathways to improve graft viability and address corneal inflammatory diseases.

## 1 Introduction

The cornea is an avascular and transparent tissue which is essential for structural stability and visual transparency necessary for sight. Although its central zone lacks a direct blood supply or lymphatic vessels, cornea has a uniquely specialized immune environment that provides successful protection against infection and reduces inflammation-related opacification. Immune privilege is sustained by a combination of various factors. These include physical barriers, immune checkpoint molecules, soluble inhibitors, and regulatory pathways that work together to limit excessive immune activation (1–4). Consequently, these processes establish an unstable equilibrium, balancing immune inactivity with the need for protection against invading pathogens. Nevertheless, these incomplete alterations may result in detrimental inflammation, scarring and even blindness.

**Figure 1:**
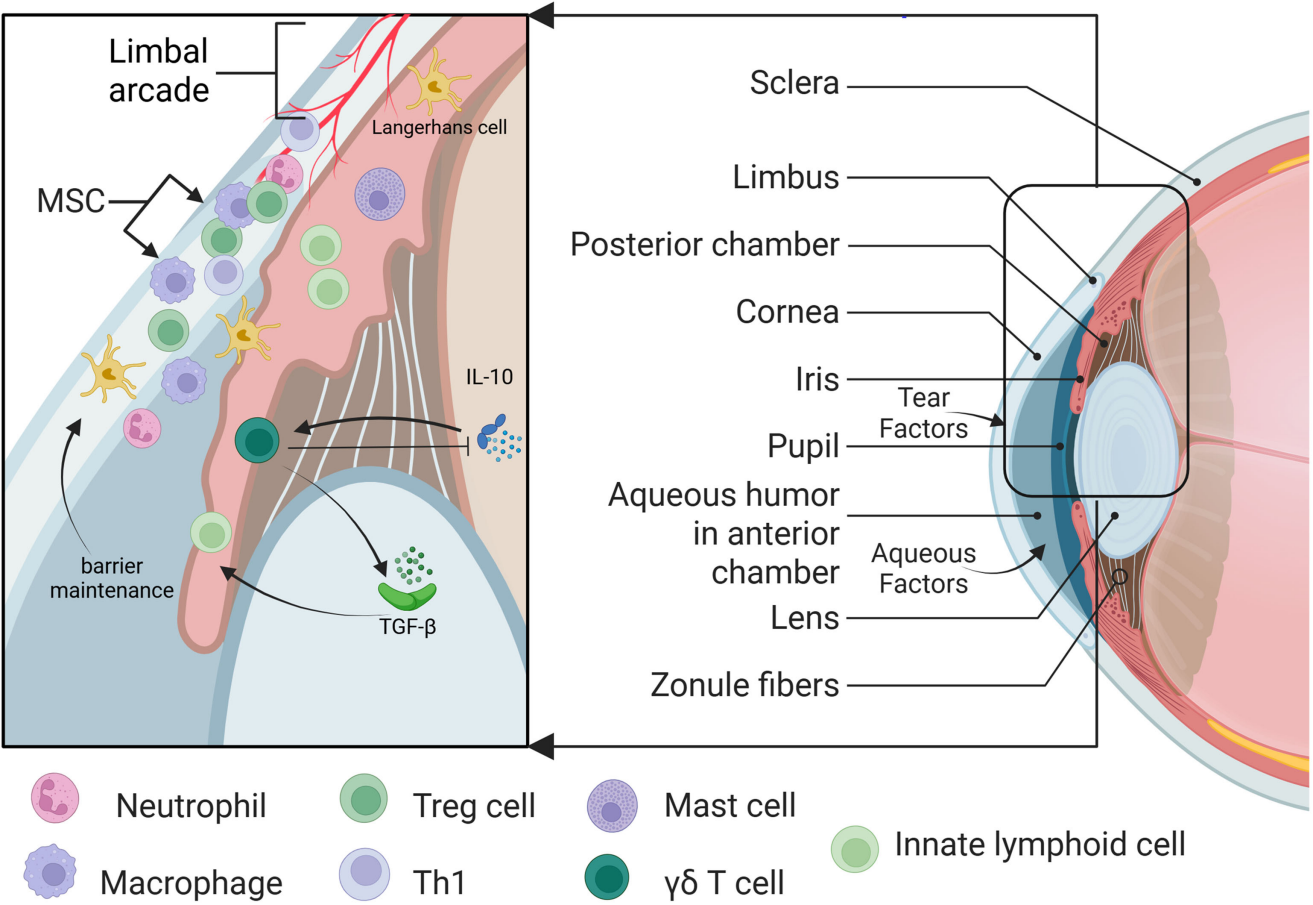
Distribution of immune cells and immune privilege at the corneal-limbal surface This figure shows the anterior part of the eyeball with highlighted cornea, Limbus and limbal vascular arch. The central area of the cornea is vascular-poor and presumably has an immune privilege on the Limbus. However, it is rich in blood vessels and immune cells, in particular neutrophils, macrophages, Th1 cells and regulatory T cells (Tregs) are concentrated in the limbus. These cells play an important role in both innate and adaptive immunity and support immune monitoring, the formation of immune tolerance and the regulation of inflammatory reactions. Mesenchymal stem cells (MSCs) in the limbus are also involved in the regulation of the immune response. This regulatory function is achieved through a variety of cell-cell interactions and cytokine signaling pathways. This regulatory function is achieved through a variety of cell-cell interactions and cytokine signaling pathways.

In a healthy cornea, resident and infiltrating immune cells, soluble mediators, and humoral factors work together to protect the cornea. The developments in immunophenotyping and imaging techniques have highlighted the functional specialization and diversity of immune cells in the cornea, including Langerhans cells, macrophages, mast cells, Gamma delta (γδ) T cell lymphocytes, and innate lymphoid cells (5–7) (**Figure 1**). All these cells are involved in monitoring pathogen, maintaining the epithelial barrier, healing the wounds, and regulating stromal integrity while immunosuppressive cytokines such as IL-10 and TGF-β, along with humoral components from tears and aqueous humor, generate an immunoregulatory condition (3, 4, 8). If this balance remains, the eyes work normally, which makes vision better and the eyes less likely to contract infections. But infections, autoimmune diseases, or surgical procedures like corneal transplants can disrupt this fine balance and make the immune response harmful.

**Figure 2:**
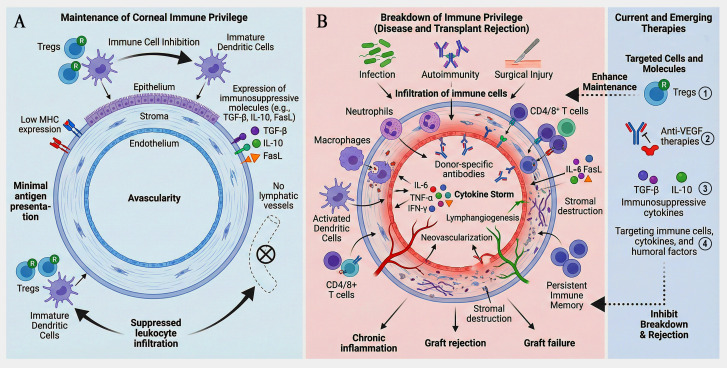
**(A):** Cellular and molecular mechanisms preserving corneal immune privilege: The cornea maintains its immune privilege via anatomical barriers, lymphatic vessels, and local immune regulatory mechanisms involving corneal epithelial and endothelial cells, tolerogenic immature dendritic cells, regulatory T cells (Tregs), and immune-suppressive mediators (TGF- b), IL-10 and FasL). These processes inhibit leukocyte infiltration, immune activation and keep the cornea transparent. **(B):** Breakdown of immune priviledge during transplant rejection and therapies. Infection, autoimmune dysregulation, or corneal transplantation can trigger immune responses that lead to neovascularization, stromal damage, and persistent immune memory. In transplantation, both cellular immunity and humoral responses, such as donor-specific antibodies, play role in endothelial rejection and graft failure. Emergent therapies targeting immune cells: Tregs and active VEGF therapies (1, 2), immunosuppressive cytokines: TGF β and IL-10 (3) and humoral factors (4) improving corneal disease and transplantation outcomes are also highlighted.

Persistent ocular surface inflammation, Infectious keratitis and alloimmune reactions can lead to neovascularization, damage to the stroma, and long-lasting immune memory. Consequently, these factors can result in enduring ocular complications or the failure of transplanted grafts (6, 9, 10). In corneal transplantation, endothelial rejection and the long-term success of grafts depend on several factors. Responses may include antibodies which are donor specific, as well as immune reactions between cells and fluids. These elements work together in a balanced environment, which is crucial for maintaining clear vision and a strong defense against infections (5, 9, 10) (**Figure 2**). With advancement in corneal immunobiology, effective therapies must target the complex interactions among immune cells, cytokine networks, and humoral factors (11). This review emphasizes the relevance of both cellular and humoral immune components in the mechanisms of corneal conditions and also discusses methods to improve the results of corneal transplants.

## 2 Resident innate immune cells of cornea

### 2.1 Dendritic cells

The main antigen-presenting cells are Dendritic cells (DCs) that are crucial for coordinating innate and adaptive immune responses (12, 13), adequate to trigger an immunological response migrating from the periphery to the lymph node, and present the antigen to T lymphocytes (14). Traditionally, observed as an immune-privileged tissue having limited antigen-presenting ability, the cornea is now understood to contain a well-structured and heterogeneous network of bone marrow-derived DCs in corneal stroma (15). Research on CD11c-EGFP transgenic mice along with immunohistochemistry have shown a remarkable distribution of DC populations throughout the epithelial and stromal layers of the non-inflamed cornea (16). Majority of immature CD11c^+^ DCs are primarily located in the basal epithelial layers close to the basement membrane. The long MHC class II–positive dendrites are found towards the apical surface to sample external antigens. Density of immune cells reduces from the outer edge to the central portion of cornea. This gradient is related to the increased immune response at the limbal region. The deeper stroma displays an extensive network of CD11b^+^ myeloid cells that support innate immunity and tissue homeostasis. Underneath these epithelial DCs, there are CD11c^-^CD11b^+^ macrophage-like cells that express low levels of MHC class II molecules (13, 16). This framework establishes a functional hierarchy. Epithelial dendritic cells (DCs) initiate an immune response after acquiring the antigens. In contrast, stromal myeloid cells have broader roles that include maintaining physical barriers and monitoring the immune system (16, 17).

Recent advancements in single-cell technologies have revealed a more complex diversity within cells, leading to the identification of previously unknown subpopulations (18). The principal types of DCs within the human body include conventional dendritic cells (cDCs), plasmacytoid dendritic cells (pDCs), monocyte-derived dendritic cells (moDCs), and Langerhans cells. These cell types are distinguished by their molecular signatures their developmental pathways, and their specific functions within the immune system. This variability is evident in the cornea, where each subset influences the immune balance (19). Under normal physiological conditions, most of the corneal DCs show an immature, tolerogenic phenotype. This phenotype has low expression of co-stimulatory molecules and the ability to support regulatory T cells (Tregs). These cells are primarily located in the limbus and periphery of ocular region, where they assess environmental antigens and maintain immune quiescence, thereby avoiding unnecessary inflammation that could compromise corneal clarity (20, 21).

**Figure 3:**
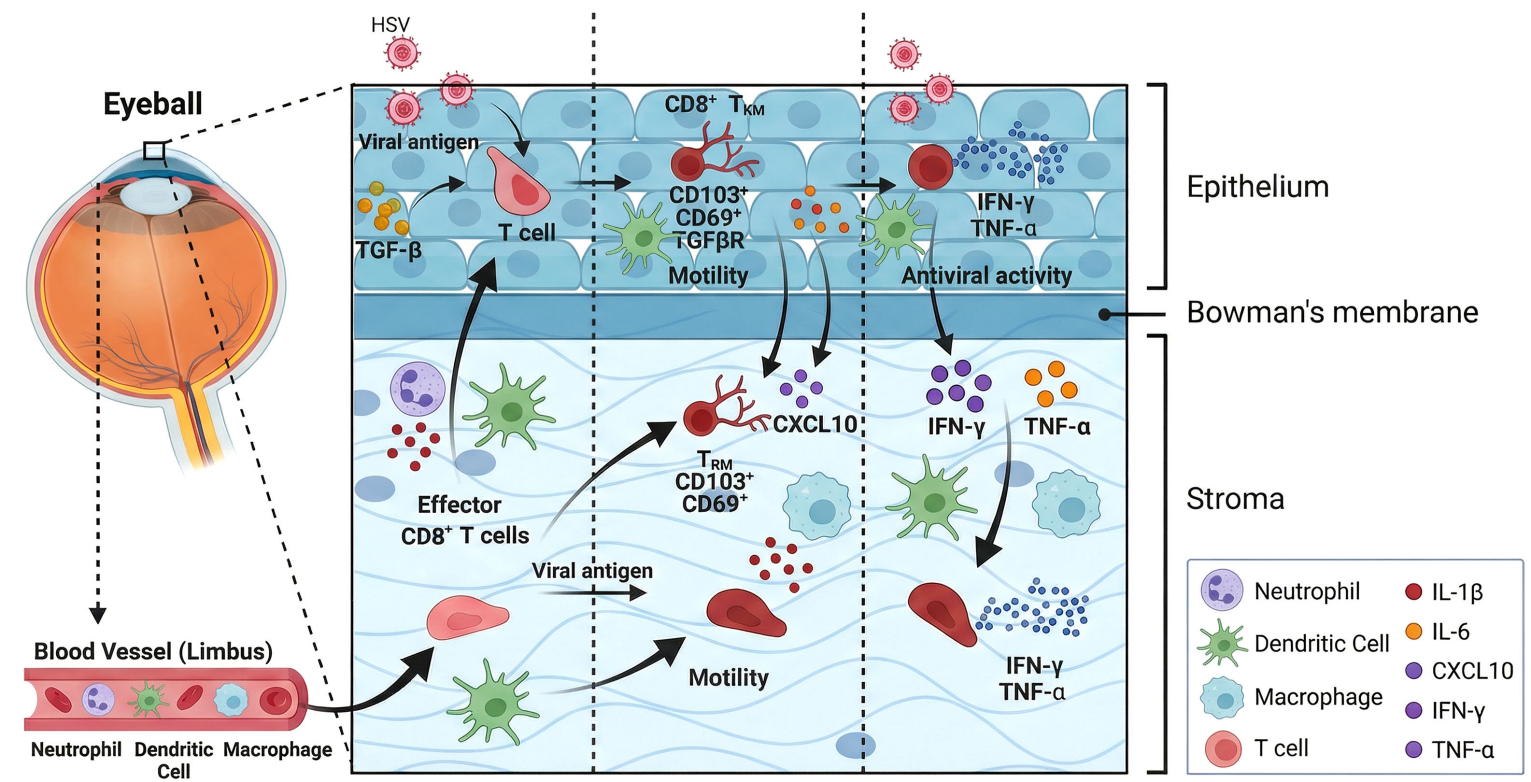
Tissue-resident memory CD8^+^ T cells contribute to corneal immunity and transplantation. During primary infection, CD8^+^ T cells are drawn from the limbus where they differentiate into CD103^+^CD69^+^ tissue-resident memory T cells in the corneal epithelium and stroma due to the impact of local signals like TGF-β. These cells endure long-term and rapidly produce IFN-γ and TNF-α when exposed to secondary antigens, exhibiting localized immune protection. Although crucial for antiviral protection, persistent initiation may interrupt corneal immunity and impact inflammation and graft survival after corneal transplantation.

Corneal DCs undergo rapid maturation after infection, mechanical damage, or inflammation, The maturation process involves upregulation of MHC class II and co-stimulatory molecules such as CD80 and CD86, an increase of cytokine production, and migration to draining lymph nodes. In this situation, the naive CD4^+^ and CD8^+^ T cells are activated, introducing strong effector responses (22, 23) (**Figure 3**). The characteristics of the activated T-cell response are greatly influenced by the local cytokine environment; DCs can either promote the expansion of pro-inflammatory effector T-cells or aid in the differentiation of Treg to reestablish immune balance. This functional adaptability helps DCs in keeping a right balance between tolerance and immunity in the cornea (24, 25).

Chronic inflammation, autoimmune processes, or transplantation can disrupt this balance through interference of DC signals or their spatial arrangement. Aberrantly activated or improperly positioned DCs may induce excessive T-cell activation, perpetuate corneal inflammation, promote neovascularization, or aid in alloimmune graft rejection (26, 27). In corneal transplants, the maturation and migration of resident DCs are important key events that influence the graft survival (28).

Diabetes mellitus (DM), a chronic metabolic disorder, is characterized by elevated blood sugar and high blood pressure, and it also affects the function of eye DCs (29). Diabetic corneal neuropathy (DCN), a common but often overlooked eye condition, is frequently misdiagnosed (30, 31). Chronic metabolic diseases induce systemic inflammation and oxidative stress, thereby damaging corneal nerves. Consequently, epithelial repair mechanisms are compromised, and immune responses are altered. These changes affect the DCs of the cornea and influence their number, shape and maturity. Therefore, a connection between corneal nerve damage, metabolic diseases and especially diabetes is suspected. The general state of health directly affects the DCs of the eye (32–34).

To understand immune-related corneal diseases, a deeper understanding of the function of dendritic cells is necessary. Researchers are investigating how these cells develop, organize, and adapt in both healthy and damaged eyes. New findings are providing insights that could help us better understand diseases and their treatments, with the goal of restoring immune balance and protecting vision (16, 20, 35, 36).

### 2.2 Corneal macrophages

Corneal macrophages are immune cells that can adapt according to the situation (37). These cells assist in forming blood and lymphatic vessels while the body is recovering during inflammation, or when formation of blood vessels is inhibited during development (38).

These functions are related to certain types of tissues and regulate neovascularization and formation of lymphatic vessels in the cornea and skin following an injury. As a result, this process affects the outcome of healing corneal wounds, organ rejection, and abnormal angiogenesis in the skin (38). Macrophages are very important for both wound healing and causing inflammation. They can shift between two distinct states: the pro-inflammatory M1 condition and the anti-inflammatory or pro-repair M2 state. This alteration is contingent upon their surrounding environment (39, 40).

Macrophages, derived from either embryonic progenitors or monocytes within the bone marrow, exhibit specialized activities in many tissues, e.g. Langerhans cells in the skin, Kupffer cells in the liver, and microglia in the central nervous system. M1 macrophages are the first cells to respond to tissue damage. They release cytokines like IL-1β and TNF-α to recruit neutrophils to the affected area (39).

M2 macrophages, on the other hand, release IL-10, which is an important anti-inflammatory cytokine that reduces inflammation and helps the extracellular matrix repair itself and tissues grow. Signaling mechanisms, involving NF-κB and STAT proteins, influence the actions of macrophages. In contrast, M2 macrophages can hinder wound healing and worsen chronic inflammation (39).

A comprehensive understanding of these mechanisms elucidates the potential of macrophage therapies that target cytokine regulation, metabolic alterations, and macrophage subpopulations, which could be beneficial for wound healing and the management of inflammatory diseases (41–43). Therapeutic strategies that target macrophage polarization or specific tissue subsets offer a promising way to control blood vessel formation and manage chronic inflammatory or degenerative diseases (38, 43, 44). The equilibrium between tissue-resident and monocyte-derived macrophages is of paramount importance. Reduction in either of them can hinder wound healing and even cause uncontrolled inflammation. Maintaining a balance between tissue-resident and monocyte-derived macrophages can impede wound healing and potentially lead to dysregulated inflammation. Tissue-resident or monocyte-derived macrophages engage with the autonomic nervous system (ANS) to regulate neuroimmune responses. Both these macrophage populations can identify and respond to neurotransmitters, via sympathetic and parasympathetic signaling pathways, thereby regulating inflammation and tissue homeostasis. The connection between the nervous system and the immune system is becoming increasingly important in understanding both neuroinflammation and neurodegeneration (43, 45).

While inflammation is indispensable for pathogen clearance, its regulation is important to avert tissue injury and facilitate recovery, a process in which macrophages play a key role. Due to their ability to acquire different functional states, tissue-resident macrophages also use various receptor expressions and initiate both inflammatory and pro-resolution responses for detecting evolutionary conserved environmental signals. They react to pathogen-associated molecular patterns (PAMPs) or damage-associated molecular patterns (DAMPs) by mounting strong inflammatory signaling and activating adaptive immunity, which guarantees an effective and sustained host defense (39). These cells are crucial for resolving inflammation and restoring balance after a threat. Recent studies using fate mapping and single-cell RNA sequencing have shown that different macrophage groups come from different embryonic sources. These groups then perform specific functions in response to changes in their environment. For example, after corneal injury, resident macrophages are quickly replaced by cells that come from monocytes. Local cytokine signals, which can either amplify or diminish inflammation, affect these cells. This suggests that treatments designed to help macrophages resolve inflammation could be beneficial. Specific approaches have demonstrated the capacity to mitigate inflammation, bolster the body’s inherent repair mechanisms, and restore tissue homeostasis (39, 46).

### 2.3 T cells

T cells are an important component in adaptive immune response against pathogens and maintenance of the immune balance within the cornea. CD4^+^ T helper (Th) cells differentiate into subcategories such as Th1, Th2, Th17, and regulatory T cells (Tregs), each with distinct immunological functions. Interferon-gamma (IFN-γ) is produced by Th1 cells that improves macrophage activation and cell-mediated immunity against intracellular pathogens (47, 48). In contrast, Th2 cells secrete IL-4, IL-5, and IL-13. These cytokines support antibody-mediated immunity by increasing antibody production and activating and differentiating B-cells, which helps with tissue repair. Th17 cells, which release IL-17, they are key drivers of inflammation against extracellular pathogens but are also implicated in autoimmune disorders and corneal graft rejection (49, 50).

T cells are a crucial component of adaptive immune defense in the cornea. They develop in the thymus into CD4^+^ helper and CD8^+^ cytotoxic cells that further differentiate into effector (Th1, Th2, Th17, and regulatory Tregs) and memory populations when exposed to antigens (47, 51). Their development is regulated by coordinated transcriptional and epigenetic mechanisms, enabling diverse functional responses during acute infection, chronic disease, tumors, and autoimmune conditions. Specialized subsets, including γδ T cells, additionally contribute to tissue surveillance and rapid host defense, while dysregulated T-cell activity can lead to autoimmune pathology (5, 47, 49, 52, 53).

Tregs are fundamental for maintaining immune homeostasis and inducing tolerance in naïve T cells even during inflammation, and thereby, preventing excessive immune responses to self-antigens and pathogens. They suppress immune activation through multiple mechanisms, including the secretion of IL-10, TGF-β, and IL-35, which inhibit effector T-cell proliferation and cytokine release, along with CTLA-4-mediated downregulation of costimulatory molecules (CD80/CD86) on antigen-presenting cells. Elevated CD25 expression enables Tregs to utilize IL-2, depriving effector T cells of this essential growth factor. These combined mechanisms establish and sustain peripheral tolerance, thereby preventing autoimmunity, chronic inflammation, and transplant rejection (54). Within the corneal tissue, Tregs serve an essential function for preserving local immune tolerance and averting inflammatory suppression, collaborating with CD4^+^ T-cell subsets, CD8^+^ cytotoxic cells, and γδ T cells to safeguard the ocular surface integrity. The essential role of these cells in peripheral tolerance and corneal immunity continues to be a pivotal element in the development of novel immunotherapies for both ocular as well as systemic inflammatory or autoimmune disorders (55–57).

Cornea can generate tissue-resident memory T (TRM) cells following its interaction with a pathogen. In murine ocular infection models, intravital two-photon microscopy has shown that CD8^+^ T cells go into the cornea and change into motile tissue-resident memory (TRM) cells and quickly respond when they come across an antigen again. For CD103^+^ TRM cells to develop, there must be antigens and TGF-β in the corneal microenvironment (6). Recent complementary imaging experiments in humans have demonstrated the existence of analogous motile immune cells within the healthy cornea. This observation indicates the presence of TRM-like populations that function as initial immune surveillance system cornea can produce localized protective memory, even though the eye is immune-privileged. This mechanism enhances pathogen readiness while preserving tissue integrity (6, 35, 58).

### 2.4 Natural killer and natural Killer T cells

Natural killer (NK) and Natural Killer T (NKT) cells are primarily found within the corneal limbus. These cells frame rapid responses to infections, thereby contributing to the regulation of localized inflammation. NKT cells support ocular immune privilege by promoting ACAID (anterior chamber associated immune deviation) tolerance, while NK cells balance cytotoxic and regulatory functions through activating and inhibitory receptors such as KIR, NKG2D, and CD16 (59, 60). Through CD1d-mediated lipid antigen recognition, NKT cells coordinate immune responses without compromising corneal integrity, and while intraepithelial lymphocytes activate DCs during dry eye to initiate Th17-driven inflammation (59, 61).

Due to the limited ability of eye regeneration, immune-mediated impairment can severely impede vision, making immune privilege status essential for suppressing excessive innate and adaptive immune responses. NK cells play a role in maintaining this balance but also exhibit context-dependent roles in ocular disease. They exacerbate herpes stromal keratitis by promoting neutrophil infiltration, yet aid in clearing *pseudomonas* keratitis through IFN-γ–dependent activation of neutrophils (8, 59, 62). Intraocular NK cell activity varies across different conditions; they may either support inflammation or promote its resolution. NK cells can eliminate uveal melanoma cells *in vitro* through identifying them as abnormal tumor cells, their activity can also be reduced by BMP4 tumor-derived signals, local immunosuppressive factors such as MIF (Macrophage migration inhibition factor) and TGF-β inhibit NK-mediated tumor surveillance within the eye (63, 64).

Following epithelial abrasion, NK cells rapidly migrate into the limbus and stroma reaching a peak around 24 hours through chemokine pathways involving CXCR3 and CCR2. Their recruitment is impaired in mice lacking ICAM-1, CD11a, CD11b, or γδ T cells. Inhibiting NK cells via NKG2D results in excessive neutrophil accumulation, delayed epithelial closure, and disrupted corneal nerve regeneration, indicating that NK cells regulate harmful inflammation and promote wound healing (59, 61). Adoptive transfer experiments have validated further that active NK cells mitigate neutrophil-mediated damage and facilitate tissue repair (65).

Differences in NK cell regulation due to age also affect corneal transplantation outcomes. In very young recipients, increased NK-cell infiltration and elevated IFN-γ production, alongside reduced Treg frequency and weaker Treg-mediated suppression, drive accelerated allograft rejection. NK-cell depletion, or transfer of adult Tregs, enhances graft survival, underscoring that an interplay between NK cells and Tregs can influence transplantation outcome (66, 67).

Collectively, NK and NKT cells serve as key modulators of ocular immunity, maintaining immune privilege, antimicrobial defense, inflammation, and wound healing. Their diverse roles across infection, injury, and transplantation highlight these cell types as promising targets for therapeutic modulation in ocular surface and intraocular disease (59, 68).

### 2.5 Neutrophils

Neutrophils play an essential role in safeguarding the cornea. They are rapidly recruited to the cornea where they phagocytose invading pathogens and release reactive oxygen species and enzymes to eliminate microbes during infection in an environment constantly exposed to pathogens, mechanical stress, and environmental insults (69, 70).

While neutrophils play a crucial role in combating infections, their overactivation or prolonged activity can precipitate uncontrolled inflammation, thereby damaging tissue, inducing corneal ulcers, and resulting in scarring within the stroma (71). Neutrophils, previously viewed as a homogenous population, are now acknowledged as a highly heterogeneous and adaptable component of the innate immune system. Their maturation, surface markers, and functional polarization can be modulated by environmental signals. Th17 cells produce IL-17, a cytokine that exacerbates inflammatory responses and inhibits tumor development through the activation of inflamN1 neutrophils. Conversely, N2 neutrophil phenotypes promote tumor growth, and reduce inflammation (72). Advances in single-cell and transcriptomic technologies have revealed multiple neutrophil subsets that play distinct roles in host defense, inflammation, tissue repair, and fibrosis. Their plasticity is influenced by intricate signaling, transcriptional, and epigenetic programs, enabling rapid adaptation across physiological and pathological contexts (70, 73).

Neutrophils function alongside epithelial cells, fibroblasts, NK cells, macrophages, and DCs to preserve corneal immunity. However, excessive or prolonged neutrophil recruitment contributes to corneal ulceration, stromal scarring, and loss of transparency (70). Transcriptomic analysis on tissues derived from patients with corneal infections consistently revealed elevated expressions of neutrophil recruitment and activation genes, correlating with higher tear cytokine levels and increased disease severity. Neutrophil extracellular traps (NETs), while useful for immobilizing pathogens, can cause tissue injury and fibrosis if not adequately regulated or removed (70, 74).

Neutrophils influence more serious ocular pathologies, including age-related macular degeneration, diabetic retinopathy, and optic neuropathies, where formation of NET and release of pro-inflammatory mediators contribute to neuroinflammation and retinal damage. Their infiltration and activation may promote disease through degranulation, NETosis, and autophagy-related pathways (74, 75).

Neutrophils play a critical role in corneal repair; their absence hinders both epithelial and nerve regeneration while disrupting key genes such as MMP9 and EGF that are closely linked with neutrophil activity. Neutrophils contribute to corneal repair via various mechanisms, MMP9 and EGF being key players. Consequently, treatments aimed at modulating MMP9 activity might accelerate corneal healing and mitigate the adverse consequences frequently observed during the repair phase (76).

Currently, researchers are exploring drug-based strategies to control how neutrophils work. The goal is to treat a wide range of diseases, including inflammatory conditions, viral infections, immune system disorders, kidney problems, and cancer (73, 77). These new methods could have effects that go beyond the cornea, potentially leading to new treatments for various health problems.

The use of corticosteroids, cytokine inhibitors, complement blockers, and protease inhibitors has significantly improved our understanding of how neutrophils work. In addition, the advancement of targeted therapeutics is facilitated by sophisticated treatment modalities, such as inhibiting NETosis, diminishing reactive oxygen species (ROS), employing migratory inhibitors, and strategies to enhance neutrophil motility. In contrast, these techniques face significant challenges regarding their specificity and the complex interactions within the immune system (74, 78). Cutting-edge technologies that amalgamate diverse omics methodologies that reveal substantial differences in neutrophils. Therefore, it is essential to devise methods that clarify the signaling mechanisms that govern their mobility, activation, and immune-regulatory roles (79). Neutrophils, once thought to be a single, short-lived player of the innate immune system, are now known to be quite diverse. These cells have different characteristics and surface features, and they perform a variety of functions. They can change their maturation, polarization, and density, which shows that they can adapt to their surroundings.

They help the body fight infections and control inflammation, heal tissue, change the immune system, and promote some viral infections and cancers. This diversity is due to intricate signaling, epigenetic and transcriptional mechanisms (80). This knowledge will be useful for developing targeted, neutrophil-based therapies for treating ocular surface disease, corneal infections, retinal degeneration, and eye cancers in humans and other species of animals while minimizing collateral tissue damage (69, 73).

### 2.6 Cytokines

Cytokines coordinate immune responses in the cornea. Pro-inflammatory cytokines such as IL-1β, IL-6, TNF-α, and IFN-γ are rapidly elevated during infection or injury, aiding in leukocyte recruitment and activation, leading to pathogen clearance. In patients affected with corneal infections, increased levels of IL-1β, IL-6, and CXCL8 (IL-8) correlate with severity of disease and neutrophil infiltration (81). This process stimulates resident corneal and conjunctival cells to produce chemokines that further amplify the inflammatory cascade. Anti-inflammatory cytokines, such as IL-10 and TGF-β, are crucial for resolving inflammation and promoting tissue repair. IL-10 inhibits the production of pro-inflammatory cytokines and reduces antigen presentation, while TGF-β promotes immune tolerance and extracellular matrix (ECM) production. IL-10 is produced by Tregs, macrophages, DCs, and B cells, while TGF-β arises from Tregs, macrophages, fibroblasts, platelets, and epithelial cells (52, 82). The levels of the pro-inflammatory cytokines (IL-1, IL-6, and TNF-α) from ocular epithelial cells determine the outcome of corneal immune responses and the likelihood of tissue damage or scarring. Cytokines are crucial for wound healing in the cornea. After injury, a carefully controlled sequence of cytokine expression directs immune cell recruitment and activation, epithelial cell movement, and tissue remodeling. Disruption of this balance, such as excessive or prolonged production of pro-inflammatory cytokines, can hinder healing and potentially lead to chronic inflammation or fibrosis, thereby causing reduced vision (52, 83).

In cases of microbial keratitis, an excessive immune response can make tissue damage worse and cause vision impairment. This confirms that we need treatments that can reduce cytokine activity without making the body’s natural defenses weaker. Other cytokines, such as vascular endothelial growth factors (VEGF), mainly associated with forming new blood vessels (angiogenesis), also impact the migration of immune cells during healing in the cornea. IL-17 is essential in host defense against microbial infections. However, dysregulated IL-17 signaling has been associated with the pathogenesis of several autoimmune and inflammatory disorders, including rheumatoid arthritis, psoriasis, multiple sclerosis, Sjogren’s syndrome, and autoimmune uveitis. This excessive or dysregulated IL-17 activity contributes to allograft rejection in corneal and solid organ transplants by enhancing inflammatory cellular infiltration, cytokine release, and tissue damage (84). Corneal epithelial abrasion also triggers a rapid CCL20-driven influx of CCR6^+^ IL-17^+^ IL-22^+^ γδ T cells, which are essential for inflammation and epithelial repair. Blocking CCL20 or employing γδ T-cell-deficient mice markedly reduces γδ T-cell recruitment, and neutrophil and platelet influx, while also delaying epithelial healing. IL-22 is an important effector cytokine, as its inhibition impairs epithelial proliferation, while IL-22 promotes wound closure and inflammatory cell recruitment (85).

Disruption of the ocular surface system leads to tear film instability and hyperosmolarity, activating stress signaling pathways and initiating the production of pro-inflammatory cytokines and MMPs. This process fosters the activation and migration of antigen-presenting cells, leading to chronic inflammation (86, 87).

### 2.7 Langerhans cells

Langerhans cells (LCs) are unique antigen-presenting cells that act as immune sentinels throughout barrier tissues, establishing a dense surveillance network in the skin, oral mucosa, corneal limbus, and conjunctival epithelium. Despite being historically categorized within the DC family, LCs are currently recognized as a distinctive subset of tissue-resident macrophages originating from embryonic precursors (88), which are distinguished by their expression of langerin/CD207 and their remarkable capability for migration and T-cell activation. Their dual identity, macrophage lineage with DC like functional behavior helps them induce either immunity or tolerance based on their microenvironment. In the eyes, LCs maintain their numbers through self-renewal and are reinforced by bone marrow derived monocytes during inflammatory responses, facilitating quick adaptation to injury or infection. LC depletion or impaired LC function heightens susceptibility to inflammatory conditions, particularly periodontitis, highlighting their importance in maintaining epithelial homeostasis (5).

LCs constantly sample antigens on the ocular surface, and modulate immune response that safeguard tissue integrity. However, in pathological conditions, they can also contribute to inflammation, graft rejection, or hypersensitivity reactions. Collectively, these characteristics establish LCs as key regulators of local immunity at the epithelial interface (5, 88, 89).

LCs are recognized as a distinct group of DCs of mesenchymal origin found in the epidermis of various mammals, where they are integral to antigen processing and express Class II major histocompatibility antigens crucial in initiating allograft rejection (90). Histochemistry and microscopic studies have shown that the cells that make up the surface epithelium of the eye are similar in humans and many other animals. When the cornea is irritated, there is increased LC proliferation but their numbers are reduced by corticosteroid treatment, emphasizing their sensitivity to immune modulation (89, 91).

LCs originate from fetal liver monocytes rather than conventional DCs, with a minor input from yolk sac-derived macrophages (92). The presence of LC-like cells in the corneal and conjunctival epithelium significantly influences ocular immunity (88). The eye’s epithelial layer contains various APCs, that include both true LCs and LC-like cells that arise from monocytes, as well as inflammatory DCs. The number of these cells increases in response to various ocular conditions, such as dry eye disease or kerato-conjunctivitis sicca, infection, allergic conjunctivitis, and herpetic keratitis (93). These cells, which are derived from macrophages, retain the capacity to migrate and present antigens, akin to DCs. They are necessary for changing how the immune system works in the area, helping the body reject a graft, and making the surface of the eye inflamed (94, 95).

The functions of LCs are contingent upon their environment. Research demonstrates that these cells can augment the immunological response. LCs have a dual role, potentially attributable to the suppression of immunological responses or the augmentation of inflammation. LCs are essential for regulating the immune response in the cornea. The immune system’s functions reflect through immunosurveillance, contact hypersensitivity, and the rejection of corneal grafts. In contrast, the specific roles of these cells in eye diseases, particularly their interactions with macrophages and their ability to move like dendritic cells, are not fully understood. Therefore, further research using animal models without Langerhans cells in combination with cell line tracing is essential. Understanding fully the influence of different types of LCs on corneal immune tolerance, inflammatory response, and transplantation success is important (15).

### 2.8 γδ T-lymphocytes

γδ T cells are distinct lymphocytes abundantly found at the surfaces of gastrointestinal, respiratory, urogenital, and ocular epithelia where they act as quick responder to infections, injuries, and cellular stress. Even though they constitute a minor fraction of circulating lymphocytes, they are abundant within mucosal and epithelial tissues. These innate immune cells primarily develop from CD4^-^CD8^-^ thymic precursors. However, some early groups are now believed to come from yolk sac hematopoiesis (96–98). Unlike classical αβ T cells, γδ T cells can recognize a wide variety of ligands, although their function is still limited by classical MHC molecules, including those in the CD1 family. Their functions encompass the maintenance of epithelial cells via growth factors like IGF-1 and keratinocyte growth factor, the elimination of virus-infected cells, and the recruitment of inflammatory cells to sites of tissue injury.

Within the mucosal and ocular barriers, γδ T cells are essential for immune surveillance, tissue repair, and inflammatory responses, primarily through the secretion of cytokines such as IL-17A and IL-22, in addition to growth factors like FGF7.These cells facilitate the regeneration of the corneal epithelium at the ocular surface, bolster the immune defense against pathogens, including fungi, and alleviate allergic conjunctivitis. Furthermore, they interact with antigen-presenting cells, innate lymphoid cells, and neutrophils, thereby enhancing the localized immune response during infection or tissue damage. Recent advancement in imaging techniques and single-cell profiling have significantly enhanced our understanding of their tissue localization, diversity, and epithelial integrity (96, 99).

γδ T cells are key regulators of ocular immune homeostasis. Although mice lacking γδ T cells typically appear healthy under sterile conditions, specific strains (e.g., C57BL/10) develop spontaneous keratitis, especially in females, demonstrating that γδ T-cell deficiency disrupts corneal immune equilibrium and allows persistent inflammation (100). Understanding how γδ T cells integrate local cytokine, microbial signals, and stress factors will be important for designing targeted therapies that enhance immune functions while minimizing harmful tissue inflammation (96).

**Figure 4:**
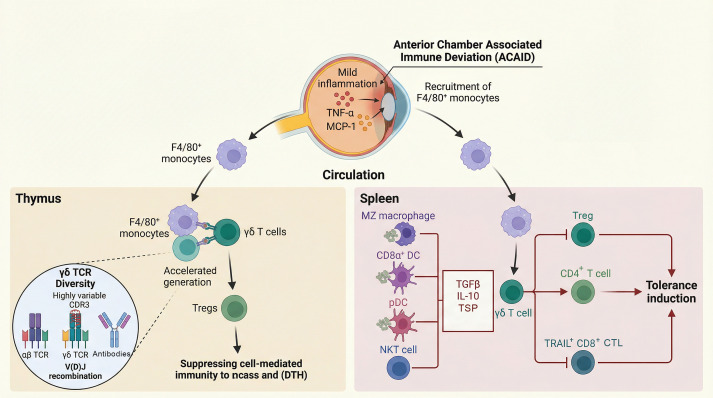
γδ T cells in ocular immune privilege and ACAID. γδ T cells play a fundamental task in ocular immune privilege and ACAID, beginning with a mild inflammation inside the eye, where molecules like TNF-α and MCP-1 attract circulating F4/80^+^ monocytes into the anterior chamber. These monocytes migrate to the thymus and spleen, where they facilitate the induction of regulatory T cells (Tregs) that suppress cell-mediated immunity and delayed-type hypersensitivity reactions, defending ocular tissues from immune-mediated damage.

γδ T cells are indispensable for maintaining ocular immune privilege due to their role in anterior chamber associated immune deviation (ACAID). ACAID initiates mild ocular inflammation, where increased TNF-α and MCP-1 promote the entry of circulating F4/80^+^ monocytes into the anterior chamber. These monocytes migrate to the thymus and spleen to promote Treg activity that inhibit cell-mediated immunity, thereby defending the eye from immune-related tissue injury (101) (**Figure 4**). They are necessary for generating Tregs that suppress delayed-type hypersensitivity responses. γδ T-cell receptors (TCRs) demonstrate remarkable diversity, with their CDR3 regions confirming the greatest variability generated by V(D)J recombination in comparison to immunoglobulins and αβ TCRs (99). Mice lacking γδ T cells or administered with anti-γδ antibodies fail to develop ACAID, highlighting their importance in maintaining tolerance within the eye (98, 100). In corneal transplantation, depletion or inhibition of γδ T cells significantly enhances the allograft rejection, confirming their protective function in graft survival. Jointly, their functional adaptability, MHC-independent activation, and competence to mediate both immunity and tolerance make γδ T cells promising therapeutic candidates for intervention in ocular surface disorders, corneal injury, and transplantation (102).

### 2.9 Humoral factors and immune privilege

The complement system plays a crucial dual role in corneal immunity, aiding in defense and mediating inflammation. It aids in pathogen opsonization, lysis and cellular infiltration. However, uncontrolled complement activation may lead to inflammation and tissue injury, particularly during transplantation or autoimmune conditions. The cornea contains complement regulatory proteins that restrict damage, and thus preserving tissue integrity (103). Complement pathway activation generates the anaphylatoxins C3a and C5a, that initiate mast-cell degranulation, increase vascular permeability, and act as efficient chemoattractant for the recruitment of neutrophils and monocytes, thereby amplifying inflammation locally (104). Simultaneously, covalent deposition of C3b on microbial surfaces (and its inactivated fragment, iC3b) opsonizes pathogens for recognition by complement receptors (CR1/CR3/CR4) on phagocytes, thereby enhancing pathogen uptake and pathogen clearance (105, 106). However, excessive or uncontrolled complement activation can lead to tissue damage, inflammation, and rejection of corneal grafts.

The maintenance of corneal immune tolerance, specifically in the context of transplantation and inflammatory diseases, depends on the equilibrium between complement activation and regulation. The complement system functions with comprehensive spatial control in ocular tissues, where C3 and C5 convertases act as the main enzyme complex regulating its activation. C3b is generated by the C3 convertase (C4b2a in the classical pathway or C3bBb in the alternative pathway) for opsonization.C5 convertase is generated, releasing C5a, a powerful inflammatory mediator, and C5b, which initiates the membrane attack complex (MAC).The mechanisms are most evident at the interface of the retinal pigment epithelium and Bruch’s membrane, where complement dysregulation markedly affects conditions like AMD. Complement regulatory proteins, including CD46 and CD55, are crucial for regulating convertase activity and maintaining immunological homeostasis in the eye (107). C3 is essential for regulating ocular inflammation and neovascularization in cases of HSV-1 keratitis. Mice lacking C3 had augmented neovascularization, heightened infiltration of monocytes, macrophages, and neutrophils, and elevated concentrations of pro-inflammatory and pro-angiogenic mediators, such as IL-1α, MMPs, CXCL1, CCL2, and VEGF-A, subsequent to HSV-1 infection. Consequently, these alterations intensify ocular opacity and tissue damage, even if both wild-type and C3-deficient mice demonstrate similar viral loads (108).

The complement system functions as a protective mechanism by regulating excessive inflammation and abnormal vascularization in HSV-1–induced ocular disease. Membrane-bound regulators, CD46 (MCP- Membrane Cofactor Protein) and CD55 (DAF) also help to preserve immune homeostasis on the ocular surface. CD46 functions as a cofactor for factor I, cleaving and inactivating C3b and C4b to reduce complement amplification, while CD55 accelerates the decay of C3 and C5 convertases, preventing excessive C3b, C5a, and MAC formation. Together, they restrict complement activation, promote immune balance, and safeguard ocular tissues within the eye’s immune-privileged environment (109).

### 2.10 Antimicrobial peptides

Antimicrobial peptides (AMPs) are primordial and evolutionary conserved humoral components of innate immunity, which play an important role in safeguarding against infections in various organisms. In addition to their wide range of microbicidal effects involving defensins and cathelicidins, AMPs influence innate and adaptive immune responses and aid in supporting tissue homeostasis (110). The ocular surface epithelium functions as both a physical barrier and an active immune interface on the eye, with its AMP network playing an imperative role in sustaining corneal health and supporting immune privilege. With the increasing prevalence of antibiotic resistance, these peptides provide important models for new therapeutic approaches and a better insight into mucosal immune regulation (52, 82). These AMPs work together with components of the tear film to maintain immune balance and the protective barrier of the eye.

AMPs perform multiple roles, including combating infections, facilitating healing, and modulating immune responses. In the presence of bacteria, corneal epithelial cells synthesize AMPs, including defensins and cathelicidins. These peptides are associated with reduced inflammation and improved healing in dry-eye disease (110, 111). They influence the immune system by regulating cytokine production and facilitating the healing of epithelial tissue. Conversely, inadequate control of these peptides may elevate the risk of infections and inflammatory disorders (112).

The dual role of AMPs highlights their importance in maintaining balance on the ocular surface (113). β-defensins (hBD1–3) and cathelicidin LL-37 damage microbial membranes and help epithelial cells move and proliferate. The cornea and conjunctiva function as physical barriers, concurrently contributing to the immune response. Ocular tissues harbor a variety of AMPs such as β-defensins (hBD-1 to hBD-4) and cathelicidin LL-37 (114). The epithelial cells are responsible for the regulation of AMPs, including LL-37 and hBD1–3. These also exhibit a variety of antimicrobial activities, which target diverse bacterial, viral, and fungal pathogens, thereby impeding pathogen colonization and safeguarding the ocular surface’s integrity (115). The levels of hBD1–3 are significantly higher in peripheral blood samples during sepsis, highlighting its role in different inflammatory responses (116). Furthermore, AMPs modulate local immune response by recruiting neutrophils and macrophages to the infection site. They also contribute to wound healing by stimulating epithelial cell migration, proliferation and restoration of the corneal barrier. AMPs protect the eye against environmental harm and microbial infiltration.

LL-37 and hBDs enhance the motility and proliferation of epithelial cells, both *in vitro* and *in vivo* (113). hBD1–3 and LL-37 compromise microbial membranes and facilitate the motility and proliferation of epithelial cells. LL-37 possesses direct antibacterial properties and serves as an imperative mediator for the innate immune system. It does this by stopping pro-inflammatory macrophage pyroptosis, boosting neutrophil antimicrobial responses like NET and ectosome formation, and speeding up the removal of pathogens (117). Collectively, these findings establish AMPs as crucial mediators of ocular surface immunity and promising therapeutic targets for infectious and inflammatory eye diseases (117, 118).

### 2.11 Secretory IgA

Tear fluid represents a significant interface of ocular immunity, specifically to corneal and ocular surface disorders. The presence of secretory immunoglobulin A (sIgA) in tears resembles the local mucosal immune activity, which is produced by the plasma cells in the lacrimal gland and conjunctival lymphoid tissue, which is subsequently secreted into the tear film, where it significantly contributes to corneal defense (119). sIgA is the principal immunoglobulin in the tear film, a dimeric protein synthesized by B lymphocytes in the accessory lacrimal glands and conjunctival epithelial cells. It acts as a key immune effector that maintains ocular mucosal surface defense and homeostasis by continuously secreting into the tear film and by inhibiting microbial adherence to the ocular surface (44). sIgA constitutes for approximately 10% of the total protein content in basal tears in humans (120, 121), and contributes to the 60–85% fraction of the total tear proteins which are composed of major protective molecules (121–124).

sIgA can neutralize pathogens and avert their adhesion to the corneal epithelium, thus inhibiting microbial colonization and infection. This protective measure ensures corneal transparency and establishes immune homeostasis at the ocular surface (125). Elevated concentrations of tear fluid sIgA can act as a non-invasive indicator of ocular surface immune activation and potential corneal disorders; sampling of tears can provide insight into underlying immunologic and structural variations in the cornea without utilizing any invasive techniques (126).

Additionally, tears have lower levels of polymeric IgA, monomeric IgA, IgG, IgM and IgE than sIgA. The concentration of different antibodies in tears may increase significantly during infection (127). The presence of sIgA in tears consequently indicates a frontline humoral immune mechanism at the ocular surface, bridging the aqueous physiological tear surrounding with the immune surveillance function of the lacrimal-mucosal system, preserving the transparency and structural integrity of the cornea by limiting inflammatory activation (128).

IgG and IgM exist in lesser quantities but are essential for systemic and adaptive immunity, especially during infections or post-transplantation. The immune privilege of cornea is shielded by attributes such as being avascular and lacking lymphatic vessels. The immunosuppressive milieu includes Transforming growth factor-beta (TGF-β), Alpha-melanocyte-stimulating hormone (α-MSH), Vasoactive intestinal peptide (VIP), the expression of Fas ligand, and programmed death-ligand 1 (PD-L1), as well as high presence of Tregs. ACAID is an essential aspect of ocular immune regulation. In this scenario, antigens introduced into the anterior chamber initiate a prompt systemic Treg response that inhibits inflammation. A breakdown in immune privilege, due to neovascularization, inflammation, or repeated transplantation, presents a greater likelihood of immune-mediated graft rejection (112, 129).

## 3 Corneal transplantation: immunity and tolerance

Corneal transplantation is one of the most successful solid tissue transplants performed, primarily because of the immune-privileged nature of the cornea, that is sustained through several mechanisms, including its avascularity, presence of immunosuppressive cytokines such as TGF-β and IL-10, and the induction of ACAID (129, 130). On the contrary, immune-mediated graft rejection continues to be the main cause of graft failure, remarkably in individuals with vascularized or chronically inflamed cornea, where immune privilege is compromised (9). Acute rejection is mainly caused by CD8^+^ cytotoxic T cells and neutrophil infiltration; however, chronic rejection is characterized by fibrosis, endothelial cell loss, and complement activation. The breakdown of immune tolerance also promotes donor-specific antibody (DSA) production, that binds to donor endothelium and triggers complement-mediated cytotoxicity and inflammation (23, 131).

In 1905, Eduard Zirm performed the first human corneal transplantation at the Olomouc Eye Clinic, located now in Czech Republic (132). Under typical homeostatic conditions, the avascular cornea conserves immune and angiogenic privilege, avoiding immune activation and vascular ingrowth (1, 130). Interference with pro-inflammatory, angiogenic, or lymphangiogenic pathways like those associated with inflamed or vascularized cornea disrupts this privilege and prompts alloimmune responses against the graft (133). Even though rejection rates remain minimal in ‘low-risk’ cases, they can escalate to nearly 70% in ‘high-risk’ recipient beds exhibiting neovascularization or chronic inflammation (134). However, disturbance in pro-inflammatory, angiogenic, and lymphangiogenic pathways results in the breakdown of immune privilege and activate alloimmune reactions against the graft (133).

Despite significant progress in microsurgical techniques, penetrating keratoplasty in high-risk patients like those with vascularized or inflamed corneal beds, or those requiring repeated transplants continues to be a significant challenge. This difficulty is primarily due to the risk of irreversible allograft rejection, since in these recipients, immune privilege is lost, making graft rejection the leading cause of corneal transplant failure. Accordingly, tailored immunosuppressive treatment directed through immunological oversight, incorporating detection of DSA, is considered one of the most promising strategies for averting rejection. While there is indirect evidence related to mechanisms of antibody-mediated rejection, the precise impact of DSA on corneal transplantation outcomes is still not fully understood (131). Assessing the utility of pre-existing or *de novo* DSA could aid in categorizing the immunological risk and promote personalized treatment for vulnerable transplant recipients (28).

Advances in lamellar and endothelial keratoplasties, including Deep Anterior Lamellar Keratoplasty (DALK) and Descemet Membrane Endothelial Keratoplasty (DMEK), have considerably reduced the rejection rates and improved visual outcomes in comparison with conventional penetrating keratoplasty (135). The corneal layer which initiates the immune response also controls the reversibility of rejection. While epithelial and stromal rejection often respond positively to topical corticosteroids (136), acute endothelial rejection necessitates more aggressive immunosuppression due to the limited regenerative ability of endothelial cells (137). Long-term immunosuppressive therapy, however, is associated with ocular and systemic side effects (138).

Recent advancement has offered more directed and safer immunomodulatory alternatives pertaining to ocular immune privilege, antigen-presenting cells (APCs), and HLA incompatibility in graft rejection (139). Clinical research continues to refine risk factors and assess outcomes among keratoplasty techniques (9, 140). Advanced immunotherapies currently provide new approaches to enhance graft tolerance. These include expansion or adoptive transfer of Tregs, modulation of cytokines by inhibiting IL-1 and TNF-α or enhancing IL-10 and TGF-β, and complement-targeted therapy (134, 141). Moreover, gene and cell-based treatments focus on the production of tolerogenic DCs and biomarker-guided personalized immunosuppression that promises better results in high-risk recipients (28, 95). Gene therapy, bioengineered corneal scaffolds, and stem-cell-based reconstruction are more effective for the cornea because to its distinct immune system and inherent regenerative capabilities (142, 143).

### 3.1 Role of corneal sensory nerves

Corneal wound healing depends on a highly coordinated epithelial neural-immune unit, where sensory neurons, epithelial cells, and resident immune cells interact in a reciprocal trophic and regulatory manner. Corneal epithelium and sensory nerves, under physiological conditions, support each other reciprocally through the synthesis of growth factors and neuropeptides that preserve the integrity of the epithelium, nerve survival, and tissue homeostasis. However, prolonged hyperglycaemia disturbs this trophic loop, leading to progressive corneal neuropathy that is characterized by reduced corneal nerve density, poorer corneal sensitivity and decreasing number of resident DCs. These modifications are conducive to the development of diabetic neurotrophic keratopathy, which is associated with significantly delayed epithelial wound healing, poor nerve regeneration and increased susceptibility to chronic epithelial abnormalities (31, 144–146).

Recent studies have demonstrated that corneal sensory nerves are not only nociceptive fibers, but they also actively control wound repair and immune response. Regulation of post-epithelial damage mediated inflammation is particularly important to sensory neurons, especially transient receptor potential cation channel subfamily V member 1 (TRPV1^+^) nerves. Depletion or pharmacological blockade of TRPV1^+^ nerves delays epithelial wound closure and results in accumulation of neutrophils, γδ T cells and pro-inflammatory CCR2^+^ macrophages, underscoring their role in maintaining a pro-regenerative microenvironment via RAMP1 and SSTR5 signaling pathways; thus, TRPV1^+^ nerves are critical regulators of corneal immune homeostasis and repair (147).

It has been shown that TRPV1^+^ neuron denervation triggers epithelial ferroptosis through p53/AKT/mTOR signaling pathway; inhibition of ferroptosis promotes wound healing, revealing a novel mechanism linking loss of nerve to poor epithelium repair. TRPV1^+^ fibres are highly enriched in the regenerating epithelium post injury and are closely spatially linked with intraepithelial CD45^+^MHC II^+^ DCs. In addition, stromal macrophages express TRPV1, further supporting the notion of direct neuroimmune interaction during tissue regeneration. Conversely, TRPM8^+^ cold sensory neurons appear to be more vulnerable to metabolic stress and are selectively lost in diabetes (148).

TRPV1^+^ fibers are abundant in the regenerating epithelium after injury and are spatially tightly associated with intraepithelial CD45^+^MHC II^+^ DCs. Furthermore, the presence of TRPV1 on stromal macrophages supports the idea of direct neuroimmune contact during tissue regeneration. Moreover, TRPM8^+^fibers recover from injury faster than Substance P (SP+) fibers, a susceptibility specific to subtypes that may help explain chronic sensory deficits and delayed repair in diabetes (149).

A disturbed neuroimmune interaction further worsens the impact of diabetic neuropathy on corneal repair. Neuropeptides like calcitonin gene-related peptide (CGRP), SP and vasoactive intestinal peptide (VIP) released from corneal sensory nerves regulate DCs, macrophages, T cells and epithelial cells to modulate immune cell recruitment, inflammatory signaling and tissue homeostasis (10, 145, 150, 151).

Local DCs and macrophages release neurotrophic factors such as ciliary neurotrophic factor (CNTF) that is crucial for maintenance and regeneration of neurons. Diabetes, reduces the number of DCs and the availability of CNTF, therefore, impairing re-innervation and prolonging corneal nerve dysfunction (10, 144, 145, 151).

Neuropeptides have distinct immunomodulatory effects, with SP generally favouring pro-inflammatory response, and CGRP and VIP favoring anti-inflammatory signaling, M2 macrophage polarization and resolution of inflammation (145, 150, 151). This idea is supported by the finding that contact between sensory neurons and macrophages in *Pseudomonas aeruginosa* keratitis promotes CGRP synthesis, which induces macrophage polarization via PI3K/AKT signaling and limits excessive inflammation (152).

Deep learning and high-resolution *in vivo* imaging studies demonstrate a close relationship between corneal nerve degeneration, impaired regeneration, and altered nerve morphology with immune cell infiltration, neuroinflammation, and disruption of corneal neuroimmune homeostasis in neuropathic corneal pain, dry eye disease, neurotrophic keratopathy, and ocular graft-versus-host disease. Quantitative assessment of corneal nerves and immune response provides valuable insights into the processes of corneal nerve pathology associated with chronic inflammation, sensory dysfunction, impaired ocular surface homeostasis, delayed healing, and progression of chronic disease (153–156). However, the role of immune cells seems to be context dependent since activated DCs can act as stimulators of nerve regeneration and repair of the epithelium after sterile injury, but can also be responsible for nerve loss and chronic inflammation in diabetic keratopathy and dry eye disease (10, 151, 157).

Neurogenic inflammation is the consequence of nerve damage following surgery, trauma, infection, aging or chronic ocular surface illness. It is mediated by the production of neuropeptides and other neuronal mediators that stimulate immune response and tissue regeneration. Pathogen clearance by dysregulated or chronic neurogenic inflammation may also lead to chronic inflammation, neuropathic pain, tissue remodeling and structural damage to the cornea (150, 158, 159).

### 3.2 Effect of corneal neovascularization

Cornea is the most transplanted and the most successful solid organ transplant (160). Allograft rejection is the most frequent cause of corneal graft failure. The development of graft rejection depends on the presence of high-risk characteristics, most notably corneal neovascularization (161). In the healthy state, the avascular and alymphatic cornea maintains immune privilege through exclusion of the vascular afferent and efferent arms of the immune reflex arc, i.e. the lymphatic vessels that traffic antigens and APCs to lymph nodes, and blood vessels that deliver effector T cells back to the tissue (135, 161–163).

Corneal transplantation has proved to be a reproducible and simple surgical model to study mechanisms regulating immunity and angiogenesis (162). Angiogenesis and lymphangiogenesis are processes that can disrupt corneal avascularity and have therefore been a focus of intensive research in recent years (163).

Traditionally, the cornea was known for (lymph)angiogenic privilege, because it was thought to be devoid of lymphatic vessels. Recent development in molecular markers has enabled the detection of lymphatic vessels in the cornea under specific circumstances (164). Injury, infection or chronic inflammation disrupt the balance between pro- and anti-angiogenic factors that favor pathological corneal neovascularization and lymphangiogenesis, with infiltration of APCs and edema that convert a privileged bed into a high risk graft environment (135, 160, 162–164). One of the strongest predictors of allograft rejection, both clinically and experimentally, is pre-existing corneal vascularization with survival decreasing from approximately 90% in avascular beds to less than or equal to 50% in vascularized, inflamed beds despite immunosuppression (135, 162, 164–166).

Topical vascular endothelial growth factor-C/D (VEGF-C/D) inhibition does not improve corneal graft survival but prevents lymphatic vessel corneal ingrowth. This significantly reduces lymphatic vessel ingrowth, but increases macrophage numbers in the cornea (166). Lymphatic vessels are particularly important since graft survival in selectively alymphatic but blood vascularized beds is comparable to low risk avascular beds, emphasizing lymphatics as the main afferent pathway for allo sensitization (135, 163, 166). Molecular control of (lymph)angiogenesis: VEGF A/C/D, macrophages and influence graft survival. Soluble VEGFR 1 as an endogenous VEGF A trap neutralizes VEGF A in the cornea, despite its presence, and in part maintains corneal avascular angiogenic privilege (160, 167). Parallel hem- and lymphangiogenesis are induced by inflammation through VEGF A, VEGF C and VEGF D; VEGF A may also directly induce lymphangiogenesis by interacting with VEGFR 2 on lymphatic endothelium or indirectly recruit VEGFR1^+^ macrophages that supply VEGF C/D (164–166, 168, 169).

Macrophage driven lymphangiogenesis plays a key role in corneal allograft rejection. Pharmacologic inhibition of macrophage VEGF C expression or NF κB signaling decreases lymphangiogenesis, APC trafficking, and rejection (164, 166, 169). Therapeutically, broad inhibition of VEGF A (VEGFR1/2 traps, aflibercept, donor pre incubation) suppresses blood and lymphatic vessels, reduces DC and T cell activation, increases Foxp3^+^ Treg responses and greatly improves high risk graft survival (160, 163, 168, 170, 171).

Selective inhibition of VEGF C/D or VEGFR 3 causes lymphatic regression and decreased Th1 priming, but survival effects are context dependent due to complex crosstalk with macrophages and other immune cells (163, 166, 168, 169, 172).

### 3.3 Gene therapy

Gene therapy, especially for the eye, has become a revolutionary method that enhances the efficacy of corneal transplants by modifying its environment. Cornea provides a unique immune-privileged status, along with its lack of blood vessels and easy access. This makes it a good candidate for targeted gene delivery and diminishes the likelihood of impacting the entire body (173, 174). Recombinant adeno-associated viruses (rAAVs) have considerable therapeutic potential due to their specific affinity for corneal epithelial, stromal, and endothelial cells, along with their minimal immunogenicity. Gene transfer using adeno-associated viruses (AAVs) aims to reduce excessive blood vessel growth and control immune responses. This approach prevents corneal neovascularization, which is a major cause of transplant rejection. This method has been demonstrated to enhance transplant functionality and longevity (173, 175). Utilizing gene editing technologies on donor corneal tissue enables accurate regulation of gene expression before transplantation, thus expanding therapeutic options. Chimeric immunomodulatory proteins, including single chain immunomodulators (scIMs), have shown promise, especially in reducing transplant rejection in patients with a higher risk.The results suggest that utilizing modified donor corneas may enhance the long-term effectiveness of corneal transplants (139).

Ex-vivo gene therapy could also be very important for making corneal transplants more personalised, which would help a wide range of patients. New gene editing techniques are also addressing corneal disorders and transplants. CRISPR-Cas9, known for its precise gene-editing capabilities, shows promise in the realm of transplant surgery. This technology could potentially reduce transplant rejection and encourage neovascularization. By enabling precise gene modification or correction, CRISPR-Cas9 can disrupt detrimental processes associated with transplant rejection, angiogenesis, and inherited disorders (176).

Preliminary investigations into transplantation indicate that manipulating VEGFA expression via CRISPR-Cas9 may mitigate or even modify corneal neovascularization, a crucial determinant of corneal integrity (75). Thus, genome editing offers significant promise for improving treatments for corneal disorders and optimizing transplant procedures (177, 178).

Advanced gene-editing techniques, including CRISPR base editors, prime editors, CRISPR–recombinase fusions, mobile genetic elements, and epigenetic editors, enable precise genomic modifications while avoiding harmful double-strand DNA breaks (179). Consequently, these developments improve the potential for substantial and enduring gene insertions, alongside the sustained regulation of gene expression, thereby significantly enhancing the feasibility of multi-gene therapeutic strategies. Gene and genome-editing therapies are anticipated to serve an essential component in regenerative corneal treatment and transplantation (173). For these therapies to be clinically applicable, increased safety, improved accuracy, and better outcomes must be demonstrated through interaction with the immune system.

### 3.4 Recombinant adeno-associated viral vectors

rAAVs have been proven effective in gene delivery for medical applications. Following more than 225 clinical trials, along with the approval of six AAV-based drugs by the FDA (180). These agents are significant given their proven functionality, adaptability to various requirements, and established safety profile. These vectors are utilized for monogenic diseases and multifactorial disorders affecting various systems, as well as in aging biology. Despite these advancements, vector-associated immune responses and ongoing challenges related to the secure and efficient delivery of genes continue to limit their therapeutic effectiveness (181). Therefore, the continued development of new production, research, and regulatory methods for AAV is essential for fully realizing its therapeutic potential.

The immune response triggered by AAVs is influenced by the vector’s design, the dose given, and how it is delivered. But, preclinical models do not predict how humans will respond, that highlights the need to develop strategies to reduce these reactions and improve therapeutic safety (182). AAV platforms offer significant potential for gene therapy in the eye, due to their established safety, their ability to target specific tissues, and the positive results seen in clinical trials, as shown by the approval of Luxturna for RPE65-related Leber congenital amaurosis. However, vector immunogenicity, limited cargo capacity, and reduced intravitreal transduction efficiency continue to be significant challenges (183).

AAVs continue to be the predominant method for addressing ocular conditions, owing to their established safety profile, capacity to infect non-dividing cells, and the possibility of sustained gene expression (175). Recent advancements in capsid engineering have yielded innovative vectors, such as AAVv128, which exhibit enhanced immunological tolerance and substantially faster transduction of photoreceptors and retinal pigment epithelium (RPE).Initial investigations utilizing murine models, rabbits, and non-human primates suggest that engineered capsid variants, such as AAVv128, outperform conventional serotypes in terms of both efficacy and tissue specificity, thereby representing a considerable advancement in retinal gene therapy (184, 185). AAV-mediated gene therapy is steadily increasing its efficacy against eye diseases. Fortunately, neutralizing antibodies are unlikely to detect AAVv128 in the body. Consequently, this characteristic promotes more stable therapeutic expression in individuals previously exposed to AAV, a factor crucial for the consistent administration of therapeutic doses (184, 186). These improvements also make AAVv128 a good choice for advanced studies of gene therapy for the eyes.

Research on the structure and function of AAVv128 indicates that its altered capsid architecture enables it to evade the immune system and facilitate intracellular mobility, thereby enhancing its ability to penetrate retinal cells (187). Initial clinical data indicate that AAV-based gene therapies demonstrate a favorable safety and efficacy profile, in the management of several corneal disorders. At the same time, early nonviral technologies show promise for treating eye disorders, particularly those that affect the retina. The tailored vectors, especially the ones that use the suprachoroidal channel, make it easier for the medicine for spreading over the retina. This strategy enables comprehensive transduction with reduced inflammation, rendering it a less invasive option compared to subretinal delivery techniques (188). The advancement in ocular gene therapy, particularly for intricate retinal disorders, and the enhancement of corneal transplantation outcomes, have resulted in the development of modified capsids such as AAVv128.These capsids provide a versatile and clinically relevant framework for secure, accurate, and efficient genomic modification (35, 189).

### 3.5 Bioengineered implants

Mesenchymal stem cells (MSC) possess considerable immunomodulatory and regenerative capabilities, rendering them beneficial for addressing immune-related corneal disorders. These encompass dry eye, transplant rejection, limbal stem cell deficit, and ocular graft-versus-host disease (oGVHD).The worldwide shortage of donor cornea has prompted the investigation of different approaches, including cell-free recombinant collagen–phosphorylcholine implants, especially for high-risk patients, thus offering immune-tolerant options for otherwise intractable corneal blindness (190, 191).

Advancements in novel substances and AI-augmented design enable personalized, clinically relevant solutions for regenerative medicine. These approaches do not cause systemic immunosuppression, promote corneal nerve regeneration, or recover ocular surface stability, regardless of cases involving vascularized corneal issues or immune-mediated disorders. This highlights the growing importance of acellular and bioengineered corneal scaffolds in addressing the shortage of donor corneas and reducing the risk of transplant rejection. Tissue regeneration relies on the orchestration of immune responses; thus, the use of immunomodulatory biomaterials, including natural polymers, hydrogels, and nanoparticle-based systems, can modulate these responses to improve tissue repair and reduce rejection (192).

Additional research validates the increasing utility of acellular and bioengineered corneal scaffolds, designed to overcome donor constraints and reduce allograft rejection (193). Recombinant collagen-derived matrices seamlessly integrate efficiently with host tissue. They encourage stromal remodeling with least inflammation. Synthetic and semi-synthetic substitutes like phosphorylcholine-modified polymers exhibit exceptional optical clarity, epithelial compatibility, and overall biocompatibility (194). Notwithstanding these improvements, application of biomaterials is still limited by unpredictable host immune reactions and the limitations of standard *in vitro* assays like ISO-10993, especially in regards of endotoxin contamination that is often overlooked.

Emerging immunomodulatory biomaterials and standardized immune-response assessment pathways offer safer, more dependable approaches to tissue-engineering. In this context, next-generation cell-free corneal implants signify a substantial advancement towards immune-compatible, regenerative alternatives for patients incompatible for conventional transplantation. Continuous developments in biomaterials and tissue engineering positions them as a transformative solution for the global management of corneal blindness (195, 196).

### 3.6 Corneal stromal stem cells

The immune privilege of the cornea is partly due to its lack of blood vessels and lymphatic vessels, which restricts immune cell infiltration. Corneal stromal stem cells (CSSCs) contribute to this immune-privileged state by modulating local immune responses (197). Recent studies suggest that CSSCs can lower inflammation and boost tissue repair, indicating their potential in regenerative therapies. Understanding more about the immunomodulatory abilities of CSSCs can lead to improved outcomes in corneal transplantations and management of immune-mediated corneal diseases (198). CSSCs offer favorable therapeutic options for corneal injuries and conditions, and their regenerative potential, directing on repair through exosomes, modulation of inflammation, and improvement in tissue transparency. Developments in epigenetic reprogramming and the need for clinical implementation highlight their role in influencing future ophthalmic therapies (191, 199).

Presently, there are several significant gaps in our interpretation of corneal immunology and regeneration. Initially, TRPM8^+^ cold-sensitive neurons, which are essential for tear secretion and preservation of the ocular surface, are not well studied, particularly regarding their regeneration after injury. While TRPM8 activators demonstrate ability for remedying dry eyes, their function in nerve repair requires further investigation; appropriate imaging tools are crucial to study TRPM8+ nerve activity in living organisms (139, 200). Additionally, the interaction between complement factor Ba elevated in rejected corneal grafts and IL-17A+ γδ T cells within the limbal area remains ambiguous. Human and murine models of dry eye disease (DED) have demonstrated that γδ T cells cause inflammation, but the combined therapeutic potential of targeting both IL-17 and γδ T cells in DED and graft rejection is still insufficiently researched. Lastly, regarding the longevity of biomaterials is concerning as decellularized bovine corneas may lose as much as 60% of glycosaminoglycans within 18 months, collectively enhancing the likelihood of structural damage and ectasia (201, 202). Innovative approaches in corneal therapy suggest promising avenues that integrate biomaterials, neuro-immunology, and artificial intelligence. Hybrid grafts that combine decellularized human amniotic membrane with CSSCs improved epithelial healing rates by 40% in Phase I clinical trials. In neuro-immune modulation, the topical application of netrin-1, a nerve guidance protein, has shown success in preclinical tests, reducing macrophage infiltration by 55% in vascularized corneas.

Recent developments in corneal treatment offer potential pathways that integrate biomaterials, neuro-immunology, and artificial intelligence. In Phase I clinical trials, hybrid grafts, which combine decellularized human amniotic membrane with CSSCs, demonstrated a 40% improvement in epithelial repair rates. In neuro-immune modulation, the topical use of netrin-1, a protein that guides nerves has proven effective in preclinical trials, decreasing macrophage infiltration by 55% in vascularized cornea this indicates that it may assist in treating inflammation and promote nerve regeneration (203, 204). Artificial intelligence is transforming the monitoring of grafts. A device created by Seoul National University uses OCT-A vascular density and aqueous humor IL-6 levels to predict corneal transplant rejection with 94% accuracy up to three months (205).

The most transplanted solid tissue is the cornea, that provides a valuable model for investigating the processes underlying immunological tolerance and immune evasion. The rejection of corneal allografts in high-risk individuals predominantly stems from increased immunological activity and the disruption of ocular immune privilege. HLA-G and other molecules like it help keep this immunological privilege by keeping immune cells from getting in and stopping new blood vessels from forming. But their functionality is impaired because they need to form dimers to work best (9).

### 3.7 Future therapeutic directions

Keeping the immune system in balance is very important for successful transplantation. If this balance is lost, then it can cause graft failure, vision complications, and significant amount of stress on the healthcare system. This is made even worse by the fact that there are not enough donor tissues around the world. Xenotransplantation, encompassing swine corneal transplants, has emerged as a prospective remedy to this deficiency, however it still presents considerable immunological obstacles.

This also determines the multifaceted mechanisms causal of corneal graft rejection, demarcating the significant immune cell populations involved, while highlighting the emerging therapeutic strategies approaches designed to foster and uphold immunological tolerance in both allogeneic and xenogeneic transplantation (2).

Corneal blindness impacts more than 5 million people globally, and despite more than 180,000 corneal transplants being performed annually, high-risk grafts still face rejection rates approaching almost 100% within a decade. With the swift progress of gene therapy in ophthalmology, one of the few areas with FDA-approved genetic therapies, the cornea has emerged as a crucial focus area due to its accessibility and immune-privileged characteristics. A major breakthrough includes the creation of a single-chain immunomodulator (scIM) engineered to replicate the full activity of the HLA-G dimer, a crucial molecule for sustaining immune privilege but restricted by its requirement for homodimerization (139).

Through AAV8-mediated ex vivo delivery, scIM significantly diminishes fibrosis, neovascularization, and inflammation in preclinical murine models and stopped rejection in a high-risk rabbit transplant model, with treated grafts remaining transparent where most controls failed. The findings suggest that gene therapy has the potential to augment immune tolerance and thereby improve transplant success. These findings underscore the critical importance of vector selection, dosage refinement, and the optimization of delivery techniques. Furthermore, immune responses exhibit considerable variability depending on the site of administration, encompassing the subretinal space, vitreous cavity, or suprachoroidal space (206).

Currently, research endeavors are concentrated on enhancing delivery mechanisms and expanding therapeutic alternatives for ocular disorders, irrespective of their etiological factors. This underscores the necessity of synchronizing safety considerations with the advancement of novel therapeutic approaches. Despite these advancements, substantial challenges and shortcomings remain, necessitating further investigation to validate the safety and efficacy of pharmaceutical applications (174).

## 4 Conclusion and future perspective

Current advances in corneal immunology have exposed how exceptionally balanced the immune system should be to preserve corneal transparency while still protecting against infection, injury, or grafted tissue. The interplay between resident immune cells (macrophages, LCs and innate lymphoid cells), infiltrating immune cells, cytokine networks, and humoral factors directs responses that determine whether the cornea remains transparent or succumbs to inflammation, neovascularization, or rejection (1, 5, 207).

Even though the cornea has an appreciable degree of immune privilege, due to its avascularity, akin to physical barriers, and dynamic immunosuppressive microenvironment that includes T regs and locally articulated immune-checkpoint molecules, this protection is vulnerable (1, 207). When regulatory mechanisms crash or are overwhelmed, for instance following injury, inflammation, or high-risk allografting, the immune activation can cause graft rejection or chronic inflammation, compromising corneal clarity and vision (2, 208).

Fortunately, novel therapeutics are being developed that prioritize healing and tolerance over inflammation. Various approaches, focusing on specific immune cell types, cytokines and immune responses that produce antibodies are required (2, 9) To improve transplant success and treat inflammatory eye diseases, strategies such as modifying local immunosuppressive signals, boosting Treg activity, and modulating production of alloantibodies that need further validation (2, 207, 209).

The cornea’s immune system, which includes both resident and infiltrating immune cells, cytokine networks, and humoral components, works together to prevent infections while keeping the cornea clear. The cornea’s ability to accept transplants better than many other solid organs is due to its immune-related advantages. These advantages include the cornea’s lack of blood vessels, an immune-suppressive environment, Tregs, and specific markers like HLA-G; however, this immune privilege could be limited. Recent research has greatly improved our understanding of how immune cells behave in the cornea. Myeloid cells, including both resident macrophages and DCs, are always present in the corneal tissue. When faced with increased danger, these cell types can change into very effective APCs, which then triggers T-cell-mediated rejection. As a result, these findings have spurred a move away from broad corticosteroid immunosuppression, favoring more targeted immunomodulatory approaches. These include tolerogenic DCs, MSC/CSSC-based therapies, and various specific methodologies designed to re-establish immune regulatory networks, rather than solely inhibiting effector responses. Other approaches use gene therapy, cellular therapies, and new biomaterials. Gene therapy has the potential to modify the transplant environment. For example, ex-vivo AAV-mediated delivery of immunoregulatory agents, including single-chain HLA-G mimetics (scIM), can reduce fibrosis, inhibit angiogenesis, and foster tolerance in adverse conditions. Acellular biosynthetic scaffolds, constructed from recombinant collagen-phosphorylcholine implants, illustrate that the risk of rejection can be lessened through the engineering of grafts that induce a reduced immune response and facilitate regeneration. Furthermore, mesenchymal and corneal stromal stem cells, along with their exosomes, demonstrate paracrine effects that diminish inflammation and fibrosis. This allows the epithelial cells to regenerate, the growth of nerves as well as clear sight Personalised treatments and combination of drugs will change the way corneal immunology and transplant function in significant ways. Improved biomarkers of rejection and tolerance, such as donor-specific antibodies, complement activation profiles, immune-cell morphologies, and tear or aqueous cytokines, will facilitate the risk assessment and enable real-time patient monitoring. Consequently, these advancements will enhance treatment protocols by transitioning from broad strategies to more tailored approaches. Future therapeutic modalities are anticipated to integrate immunological heterogeneity with gene-centric methodologies. Gene editing and the modulation of immunological checkpoints exemplify these technological applications. Furthermore, regenerative therapies, including MSC/CSSC interventions, neurotrophic support, and bioengineered scaffolds, demonstrate this favorable trajectory. However, some challenges continue to exist. The high costs and complex nature of biologics and gene therapies hinder their global accessibility, particularly for people with monocular visual impairment. A thorough evaluation in both preclinical and clinical settings is essential to reduce potential risks related to long-term expression, unexpected immune reactions, and other factors affecting the complement system. These interactions influence the efficacy and the rate of physical recovery. Investigators continue to work on the interaction of immune system and healing. The immune system and neurotrophic factors work together to speed up the body’s healing process. Understanding the neurotrophic factors and the immune system interaction is essential for developing the best treatments. The future of this field is likely to extend far beyond just preventing organ rejection. The focus will shift to developing lasting tolerance and encouraging tissue regeneration. The goal is to combine key findings about how immune cells and cytokines work with advancements in gene editing, vector design, and new biomaterials. These would substantially enhance the quality of life for countless individuals, in both human and animal populations, affected by corneal blindness and ocular surface inflammatory conditions.
